# Ultrasound-guided versus traditional method for peripheral venous access: an umbrella review

**DOI:** 10.1186/s12912-022-01077-9

**Published:** 2022-11-09

**Authors:** Carlos Berlanga-Macías, Ana Díez-Fernández, José Alberto Martínez-Hortelano, Irene Sequí-Domínguez, Alicia Saz-Lara, Diana Pozuelo-Carrascosa, Vicente Martínez-Vizcaíno

**Affiliations:** 1 University of Castilla-La Mancha, Health Care and Social Research Center, Cuenca, Spain; 2 University of Castilla-La Mancha, Faculty of Nursing, Albacete, Spain; 3 University of Alcalá, Facultad de Enfermería y Fisioterapia, Departamento de Enfermería y Fisioterapia, Enfermería, Cuidado comunitario y Determinantes Sociales de la Salud, Av. de León, 3A, 28805 Alcalá de Henares, Madrid Spain; 4 Universidad Autónoma de Chile, Facultad de Ciencias de La Salud, Talca, Chile

**Keywords:** Ultrasound, Catheterization, Vascular access, Intravenous, Landmark, Review

## Abstract

**Background:**

Short peripheral catheters (SPC) insertion technique has a high failure rate, one of the reasons why the ultrasound (US)-guided method has been proposed as a valid alternative to traditional technique in SPC insertion. This umbrella review aims to synthesize the available evidence comparing the US-guided method with the traditional method on SPC insertion in terms of effectiveness, safety and patient satisfaction.

**Methods:**

An umbrella review addressing the comparison between US-guided versus traditional method for SPC insertion in which only systematic reviews of all comparative study designs were eligible was carried out. Previous systematic reviews and meta-analyses were systematically searched in MEDLINE, EMBASE, Web of Science and Cochrane Library. Methodological quality was assessed with AMSTAR-2 tool. The quality of evidence per association was assessed using the GRADE criteria and was stablished as high, moderate, low and very low.

**Results:**

Twelve systematic reviews with a range of 75–1860 patients were included. Moderate certainty evidence supports the positive effect of US-guided method on first-attempt success rate and number of attempts. There is moderate certainty evidence that US-guided method does not reduce the time spent in SPC insertion. Low certainty evidence supports that US-guided method improves both overall success rates and patient satisfaction. Emergency department was the main hospital department where these findings were reported.

**Conclusions:**

The best current evidence indicates that US-guided method for SPC insertion is postulated as a valid alternative for both adult and pediatric population, especially in patients with difficult venous access and in hospital departments where optimal vascular access in the shortest time possible is critical.

**Trial registration:**

PROSPERO: CRD42021290824.

**Supplementary Information:**

The online version contains supplementary material available at 10.1186/s12912-022-01077-9.

## Background

Peripheral venous access devices (PVADs) are required in more than a billion hospitalized patients each year, which corresponds to 59–80% of hospitalized patients depending on the region, becoming the most used clinical invasive device [1, 2]. In the emergency department (ED), PVADs are placed in 70% patients [3]. However, PVADs insertion failure rate is between 43 to 59%, [4] a disappointing data considering the pain, care delays and infection probability to which patients are exposed [5]. Several patient-related circumstances such as chronic illness, edema, obesity or the use of injecting drugs could hinder the PVADs insertion [6]. Among the PVADs, according to international consensus standards, both the short peripheral catheters (SPC), the long peripheral catheters (LPC) and the midline catheters (MC) could be described [7, 8].

Regarding the SPC, the traditional method used for SPC insertion requires vein visualization and/or palpation from the provider [9]. Recently, the potential advantages of ultrasonography (US) guided over the traditional method in SPC insertion have been assessed [10]. In this sense, several reviews have been published to elucidate the outcomes which significantly improve when using the US-guided method in comparison with traditional method, especially in patients with difficult venous access (DVA) [11]. However, there are some methodological differences which could be responsible for the fact that, to date, not conclusive evidence is available supporting US-guided method over the traditional one [11, 12]. Likewise, there is a lack of information as to the differences between both methods in SPC-related complications [12, 13].

Umbrella reviews are a useful strategy to overcome these concerns because they assess the consistency of the evidence that the existing systematic reviews and meta-analyses provide. In our case, this methodology was used to synthesize and critically assess the evidence provided by previous systematic reviews and meta-analyses that have compared the effectiveness of US method to that of the traditional method in SPC insertion.

## Methods

This umbrella review was registered in the International Prospective Register of Systematic Reviews (PROSPERO) (Registration Number: CRD42021290824) and was adhered to both the Cochrane Collaboration Handbook [14] and the Preferred Reporting Items for Systematic Review and Meta-Analyses (PRISMA) guidelines (PRISMA Checklist, Table S1, Supplementary data) [15]. Because we are conscious that the Preferred Reporting Items for Overviews of Reviews (PRIOR) guidelines will be soon released, we have also considered most of the key aspects of methods and results reported in the majority of the overviews of reviews published so far [16].

### Search strategy

A comprehensive literature search was conducted in MEDLINE (via PubMed), EMBASE (via Scopus), Web of Science, and Cochrane Library from their inception until February 23, 2022. The following relevant terms were combined through Boolean operators: ‘Ultrasound’, ‘Echoguided’, ‘Ultrasound-guided’, ‘Sonography’, ‘Ultrasonography’, ‘Echo’, ‘Peripheral vein’, ‘Peripheral venous’, ‘Peripheral intravenous’, ‘Vein’, ‘Venous’, ‘Intravenous’, ‘Vascular’, ‘Cannulation’, ‘Access’, ‘Catheterization’, ‘Arterial’, ‘Radial’, ‘Femoral’, ‘PICC’, ‘Central’, ‘Midline’, ‘Systematic review’ and ‘Meta-analysis’. The references of the selected studies and grey literature were reviewed in order to identify additional studies. Articles retrieved were imported and managed by Mendeley reference manager. The search strategy is specified in supplementary material (Table S2).

### Selection criteria

Systematic reviews or meta-analyses comparing and addressing the effectiveness, efficiency and patient satisfaction of US-guided peripheral intravenous access versus traditional landmark method in both pediatric and adult patients were included. According to the PICO strategy tool, inclusion criteria were as follows: (a) participants: children (included small children) and adults; (b) intervention: ultrasound-guided peripheral intravenous access; (c) comparison: traditional and ultrasound-guided peripheral intravenous access method; and (d) outcome: efficiency and effectiveness, which were measured through both success and time to cannulation, type and/or number of associated complications and patients’ satisfaction (or parents satisfaction in the case of pediatrics).

Studies were excluded when: (a) involved patients with long-term central venous lines; (b) ultrasound was used to insert an arterial or central venous access, or to insert a LPC or MC; (c) the ultrasound group was compared to another different from the traditional method (such as innovative vascular access technologies, infrared devices or doppler); and (d) its design was not systematic review or meta-analysis of all comparative study designs (randomized control trials, cohort, quasi-experimental, or combinations thereof). No language restriction was applied.

### Data extraction

After screening retrieved original systematic reviews, the following data were collected: (a) author and publication year; (b) design and number of included studies in each systematic review; (c) whether or not the meta-analysis was performed; (d) total sample and participants age; (e) no exposed group: defined as traditional landmark method for peripheral intravenous access; (f) exposed group: defined as ultrasound-guided method for peripheral intravenous access; (g) DVA definition; (h) main outcomes measured (overall success, first-attempt success rate, number of attempts, time to success, patient satisfaction and associated complications); (i) professional provider and (j) hospital department.

### Methodological quality assessment

The AMSTAR-2 tool was used for the assessment of the methodological quality of systematic reviews that included randomized or non-randomized studies of healthcare interventions, or both. After assessment, the overall confidence in the results of the analyzed review may be high, moderate, low or critically low (if there are more than one critical flaw) [17].

### Evidence quality assessment

The Grading of Recommendations, Assessment, Development, and Evaluation (GRADE) criteria were applied for the assessment of the certainty of evidence of the effect of both techniques on main outcomes measured. GRADE was only applied to meta-analyses of randomized controlled trials, as recommended by Pollock and colleagues, [18] and the domains evaluated were as follows: (i) number of participants; (ii) risk of bias of trials; (iii) heterogeneity; and (iv) methodological quality of the review. Level of evidence was ranked according to established GRADE criteria as high, moderate, low and very low [18].

Two reviewers (C.B-M and JA.M-H) carried out independently the search strategy, studies selection, data extraction, and risk of bias and quality of evidence assessment. Any disagreement was solved by consensus, and, if it could not be reached, a third reviewer was consulted (V.M-V).

### Quantitative synthesis

It was not possible to carry out a meta-analysis due to the high heterogeneity of quantitative data from the previous systematic reviews, as originally planned and stated in the PROSPERO record.

### Summary findings

As a meta-analysis was not carried out, the results related to the main outcomes provided by each systematic review were graphically represented. Forest plots were used to represent estimates of the main outcomes from original studies, comparing both methods, where stated, including confidence intervals, sample sizes of each group and heterogeneity observed (I^2^). Using information provided in the included studies, the estimates were represented as relative risk or odds ratio [19], and mean difference or standardized mean difference, depending on the nature of the effect measures. Graphs were performed using STATA SE software, version 15 (StataCorp, College Station, TX, USA).

## Results

### Study characteristics

**Fig. 1:**
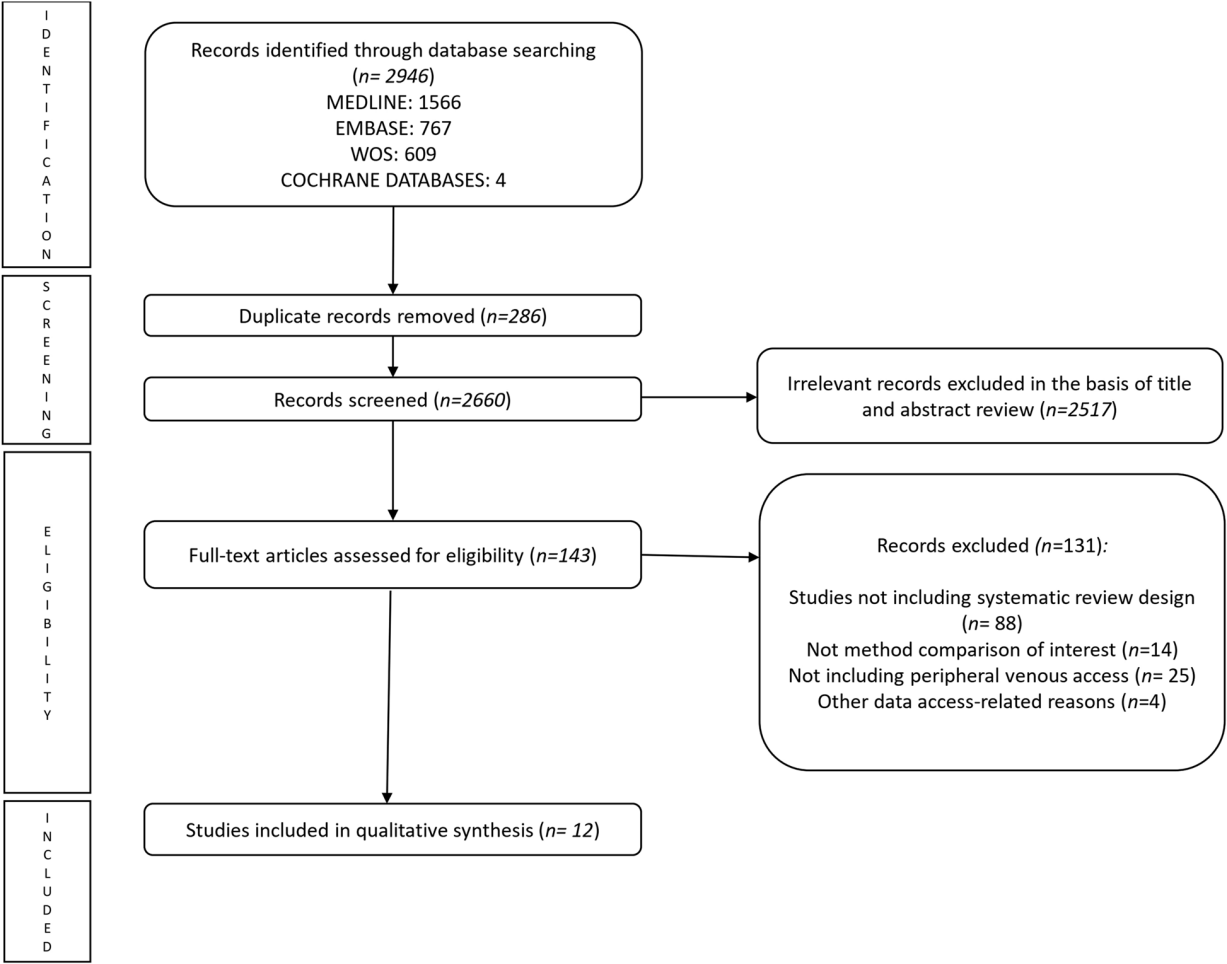
Preferred reporting items for systematic reviews and meta-analyses flow diagram of identification, screening, eligibility and inclusion of studies. WOS, Web of science

The comprehensive literature search retrieved a total of 2946 articles. From all of them, 12 systematic reviews were included in this systematic review. (Fig. 1) The detailed reasons for exclusion of full-text articles are available in supplementary material (Table S3).

The main characteristics of the included systematic reviews are summarized in Table 1. All systematic reviews were published between 2012 to 2022, and only four did not provide quantitative synthesis [13, 20–22]. The number of included studies in each review ranged from two [22] to ten [11], most of them provided data from randomized controlled trials (RCT), but four studies also incorporated data from observational studies [11, 12, 20, 23]. Likewise, the sample size of the included systematic reviews ranged from 75 to 1860 [11, 22]. Five studies included only adults as reference population; [11, 12, 20, 22, 24] both adults and pediatric population were included in four studies; [10, 13, 23, 25] and, finally, three studies included only pediatric population [21, 26, 27].

**Table 1:** Characteristics of the studies included in the systematic review

Reference	Reviewed studies		Meta-analysis	Patients		Control group	History of DVA	Outcome [ES (95% CI); I^2^]						Professional provider	Hospital department	AMSTAR score
	**Design**	**No**		**Total sample**	**Age (years)**			**Overall success**	**First-attempt success rate**	**No. Attempts**	**Time**	**Satisfaction**	**Associated complications**			
Egan et al. 2012 [25]	RCT	7	Yes	289	> 18 < 10 < 7	Standard technique	Yes	OR = 2.42 (1.26,4.68); I^2^ = NA	NA	WMD = -0.64 (0.76,-0.53); I^2^ = NA	WMD = 1.18 (-3.55,5.90); I^2^ = NA	NA	NA	Anesthetists, physicians, pediatricians and nurses	ICU, ED and trauma center	Critically low
Heinrichs et al. 2012 [10]	RCT	9	Yes	376	> 18 < 10 < 7 < 3	Traditional method	Yes	NA	NA	MD = -0.63 (-1.06,-0.20); I^2^ = 65%	MD = -0.56 (-1.14,0.03); I^2^ = 74%	NA	Arterial puncture [NA]	Anesthetists, physicians, nurses and physician/anesthetists-nurse couple	OR*, ED and ICU	Critically low
Kleidon et al. 2021 [26]	RCT	5	Yes	1029	< 18	Traditional landmark method	Yes	RR = 1.10 (0.94,1.28); I^2^ = 84%	RR = 1.60 (1.02,2.50); I^2^ = 91%	Included (No quantitative synthesis)	Included (No quantitative synthesis)	Included (No quantitative synthesis)	Infiltration, phlebitis and pain [NA]	Anesthetists	OR*, ED and surgical inpatient unit	Critically low
Liu et al. 2014 [13]	RCT	6	No	316	> 18 < 10 < 3	Traditional landmark method	Yes	Included (No quantitative synthesis)	Included (No quantitative synthesis)	Included (No quantitative synthesis)	Included (No quantitative synthesis)	NA	NA	Pediatric ED attending, fellows, investigator physicians, ED attending and residents and nurses	OR*, ED and ICU	Critically low
Parker et al. 2017 [21]	RCT	3	No	508	< 18	Traditional techniques	Yes	NA	Included (No quantitative synthesis)	NA	NA	NA	NA	Physicians	OR* and ED	Critically low
Parker et al. 2017 [22]	RCT	2	No	75	18–91	Traditional techniques	Yes	NA	NA	NA	NA	NA	NA	Physicians and nurses	OR* and ED	Critically low
Stolz et al. 2015 [23]	RCT Observational	7	Yes	NA	> 18 < 10 < 3	Traditional techniques	Yes	OR = 3.96 (1.75,8.94); I^2^ = 41%	NA	WMD = -0.50 (-1.36,0.35); I^2^ = 83%	WMD = -1.07 (-4.66,2.52); I^2^ = 75%	NA	NA	Physicians and nurses	OR*, ED, pediatric ED and ICU	Critically low
Tran et al. 2021 [11]	RCT Observational	10	Yes	1860	53 ± 5	Landmark and palpation method	No	NA	OR = 2.1 (1.65,2.7); I^2^ = 2.9%	SMD = -0.27 (-0.539,-0.004); I^2^ = 59%	SMD = -0.09 (-0.31,0.13); I^2^ = 38%	SMD = 1.47 (0.92,2.01); I^2^ = 0%	Hematoma, arterial and nerve puncture, nerve pain and infiltration [NA]	Physicians, medical student, nurses and ED technician	NA	Critically low
Tran et al. 2022 [24]	RCT	7	Yes	527	> 18	Landmark and palpation method	Yes	NA	OR = 2.09 (1.43,3.05); I^2^ = 0%	SMD = -0.15 (-0.31,0.01) I^2^ = 0%	SMD = 0.15 (-0.08,0.38) I^2^ = 0%	SMD = 0.23 (-0.66,1.11) I^2^ = 77%	Hematoma, inflammation, accidental catheter removal, obstruction, pain and infiltration [NA]	Nurses	OR*, ED and ICU	Critically low
van Loon et al. 2018 [12]	RCT Observational	8	Yes	1660	> 18	Traditional palpation and direct visualization technique	No	OR = 2.49 (1.37,4.52); I^2^ = 69%	OR = 1.82 (0.68,4.90); I^2^ = 69%	MD = 0.92 (-0.10,1.94); I^2^ = 92%	MD = 4.74 (-2.09,11.57); I^2^ = 87	MD = 32.57 (22.42,42.73); I^2^ = 0%	Pain and arterial and nerve puncture and infiltration [NA]	Physicians and nurses	OR*, ED and ICU	Critically low
Varnderll et al. 2018 [20]	RCT Non-RCT Observational	9	No	1528	≥ 16	Palpation technique	Yes	Included (No quantitative synthesis)	Included (No quantitative synthesis)	Included (No quantitative synthesis)	Included (No quantitative synthesis)	Included (No quantitative synthesis)	Hematoma, arterial puncture, pain and infiltration	Nurses	ED	Critically low
Ye et al. 2022 [27]	RCT	9	Yes	1312	< 18	Palpation technique	No	RR = 1.13 (1.01, 1.26) I^2^ = 77.5%	RR = 1.53 (1.14, 2.04) I^2^ = 84.5%	SMD = -1.93 (-3.44,-0.42); I^2^ = 98.3%	SMD = -0.46 (-1.20,0.28); I^2^ = 95.1%	NA	Infiltration [RR = 1.59 (0.99,2.54) I^2^ = 0%] and phlebitis [NA]	Anesthetists, physicians and nurses	OR* and ED	Critically low

*CI* Confidence interval, *DVA* Difficult venous access, *ED* Emergency department, *ES* Effect size, *ICU* Intensive care unit, *MD* Mean difference, *NA* Not available, *No* Number, *OR* Odds ratio, OR* Operating room, *RCT* Randomized controlled trial, *RR* Relative Risk, *SMD* Standardized mean difference, *WMD* Weighted mean difference

The exposure variable in control group of all reviews was traditional method-guided SPC insertion, defined as both standard/traditional method/technique [10, 21–23, 25] and landmark/palpation and direct visualization technique [11–13, 20, 24, 26, 27]. Only three out of ten studies did not describe if reference population had history of DVA [11, 12, 27].

Overall, the outcome variables related to the efficiency and effectiveness of US-guided method were as follows: (a) overall success, (b) first-attempt success rate; (c) number of attempts; (d) time spent for SPC insertion; (e) satisfaction of patients; and (f) associated complications to technique (pain, phlebitis, hematoma, accidental catheter removal, obstruction, arterial or nerve puncture and infiltration) [8–11, 18–25].

Finally, the professional providers participating in the included reviews were anesthetists, physicians, pediatricians, nurses, investigators, fellows and residents or students which carried out the technique in ED, intensive unit care (ICU), operating room (OR), trauma center or surgical hospitalization unit. Only one systematic review did not report the setting in which SPC was inserted [11].

### Methodological quality assessment

According to AMSTAR-2, no included studies met all the criteria included in the scale, and the compliance level ranged from 44 to 75% [10, 12, 23, 25, 26]. It should be noted that the overall confidence in the results of all systematic reviews was stablished as ‘Critically low’. On the other hand, all the included reviews met the items related to ‘PICO components’ and ‘Managements of conflict interest’. The ‘Partial yes’ option was assigned to ‘Detailed study description’ item in all the studies. (Table S4, Supplementary data).

### GRADE evidence quality

Evidence quality of the 27 associations assessed according to GRADE ranged from ‘Moderate’ (11%) [11] to ‘Very low’ certainty evidence (7.4%) [24, 25]. Twenty-two associations assessed showed ‘Low’ certainty evidence (Table S5) [10–12, 23–27]. The evidence map (Table S6) shows that the US-guided method is a protective factor related to both ‘first-attempt success rate’ and ‘number of attempts’ outcomes, with a ‘Moderate’ certainty evidence [11]. Likewise, there is no effect on the use of US-guided method for ‘time’ outcome (‘Moderate’ certainty evidence) [11].

### Summary findings

**Fig. 2:**
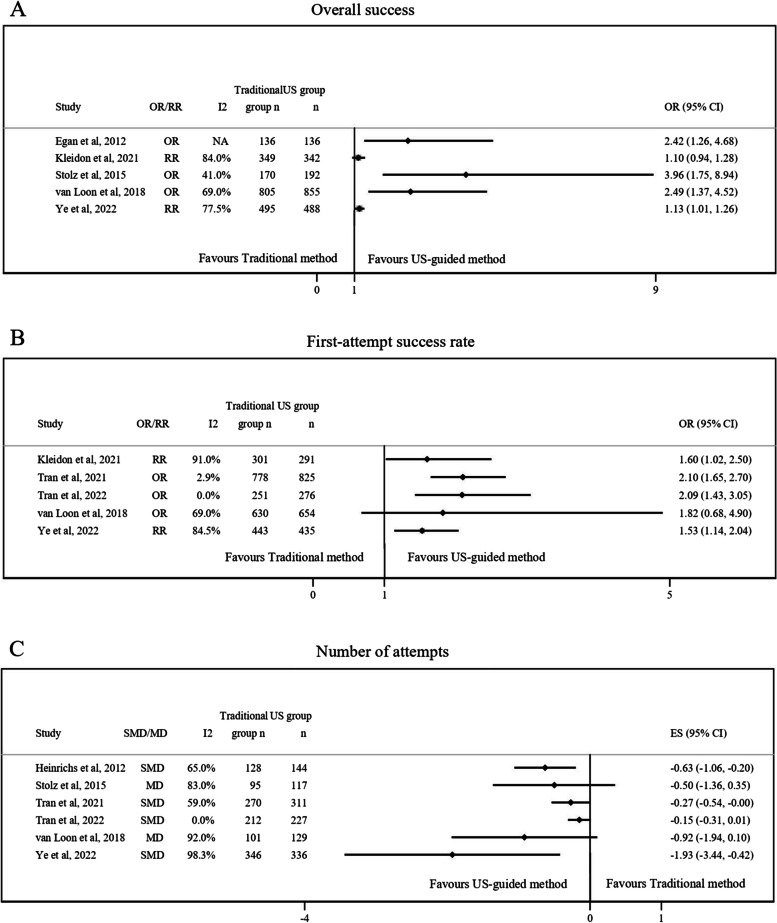
Graphical representation of data regarding (**A**) overall success, (**B**) first-attempt success rate and (**C**) number of attempts of US-guided method compared to the traditional method. CI, Confidence interval; MD, Mean difference; NA, Not available; OR, Odds ratio; RR, Relative risk; SMD, Standardized mean difference; US, Ultrasound

**Fig. 3:**
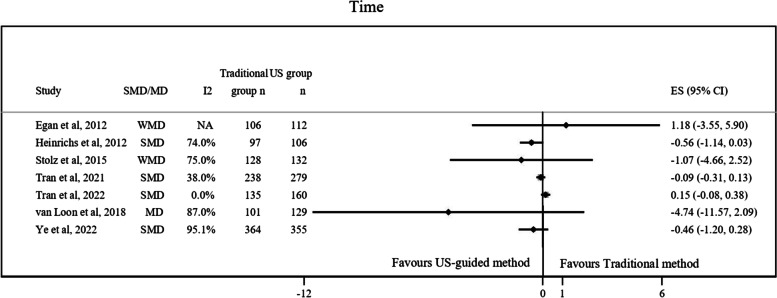
Graphical representation of data regarding time spent using US-guided method compared to the traditional method. CI, Confidence interval; MD, Mean difference; NA, Not available; SMD, Standardized mean difference; US, Ultrasound; WMD, Weighted mean difference

**Fig. 4:**
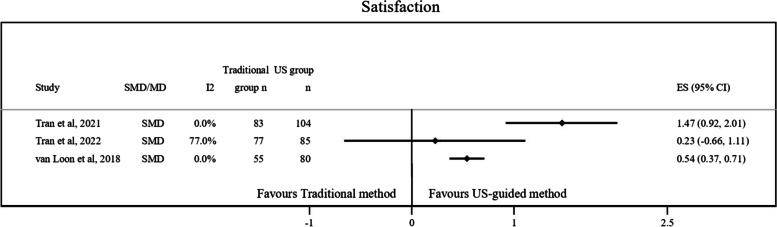
Graphical representation of data regarding patient’ satisfaction using US-guided method compared to the traditional method. CI, Confidence interval; MD, Mean difference; SMD, Standardized mean difference; US, Ultrasound

The main results of included systematic reviews were graphically pooled by main outcome variables (Figs. 2,3 and 4).

Figure 2 shows the main results related to (A) overall success, (B) first-attempt success rate and (C) number of attempts. Regarding overall success (Fig. 2A), five reviews provided data for the stated relationship, [12, 23, 25–27] and four of them found higher success in US method with respect to traditional method, ranging the RR/OR estimates from 1.13 to 3.96 [12, 23, 25, 27]. The heterogeneity (I^2^) ranged from 41 to 84%.

The findings about first-attempt success rate, for which five studies provided data, are shown in Fig. 2B [11, 12, 24, 26, 27]. Four studies [11, 24, 26, 27] found a significant positive association between US method and first-attempt success rate, ranging the RR/OR estimates from 1.53 to 2.10. The I^2^ ranged from 0 to 91%.

Figure 2C states the results related to the number of attempts. Seven studies provided data about the stated outcome, [10–12, 23–25, 27] and only three of them found that US method required lower number of attempts than traditional method, ranging the SMD/MD estimates from -0.27 to -1.93 [10, 11, 27]. The data provided by Egan and colleagues [25] could not be included in the forest plot due to a discrepancy between the effect size and confidence interval. The I^2^ observed was between 0 to 98.3%.

About the findings about time spent in SPC insertion using both methods, neither of the seven studies which analyzed the association between the method used for SPC insertion and the time spent during the technique found significant results (Fig. 3) [10–12, 23–25, 27]. The I^2^ observed was between 0 to 95.1%.

Figure 4 highlights that patient satisfaction was higher when US method was used, ranging the SMD estimates from 0.54 to 1.47 [11, 12, 24]. The I^2^ observed was between 0 to 77%.

Finally, secondary outcomes such as pain, phlebitis, hematoma, accidental catheter removal, obstruction, arterial and nerve puncture and infiltration -associated complications- were not graphically represented due to the unavailability of quantitative data. Seven studies took into account these complications, [10–12, 20, 24, 26, 27] where infiltration, pain and arterial puncture were the most frequently mentioned, and only one study reported pooled quantitative data about infiltration in pediatric patients, and a significant protective effect of US-guided method was not found [RR = 1.59 (0.99,2.54) I^2^ = 0%] [27]. Together with the infiltration, the pain reported by patients was the most frequently secondary outcome discussed [12, 20, 26]. Among all the original RCTs included in the previous meta-analyses, one RCT which included adult patients reported lower pain rates when US-guided method was used, [28] while other RCT which studied pediatric patients showed lower arterial puncture rates when US-guided method was used [29] (Table S7).

## Discussion

The synthesis of the systematic reviews and meta-analyses included in this umbrella review support that the use of US-guided method, compared to the traditional method, increases the success rates and reduces the number of attempts needed in SPC insertion in both adults and pediatric patients. Likewise, the satisfaction was higher in those groups of patients in whom the US-guided method was used, albeit with a low certainty level. Conversely, it is not clear whether the US-guided method is more efficient than the traditional one because there was not a significant reduction in the time spent in SPC insertion.

The previously available evidence regarding the effectiveness of US guided cannulation seems to be controversial. The majority of meta-analyses published to date support that the overall success, the first-attempt success, and the number of attempts rates support the use of the US guided procedure, and it could decrease problems associated to SPC insertion, such as pain, dissatisfaction or anxiety [30].

Considering that pediatrics have particular conditions and a higher failure rate, as it happens in adults, [4, 31] US is presented as a valid alternative in both populations. However, Kleidon and colleagues [26] did not find a positive effect of US-guided method in the overall success in children, as reported in four previous meta-analyses in both adult and children [12, 23, 25, 27] and in a recent study that showed an overall success rate above 90% [32]. On the other hand, the findings of Van loon and colleagues [12] are not in line with the association between US-guided method and the first-attempt success rate, which was reported with a moderate certainty level [11, 24, 26, 27] and which is supported by a recent study in patients with DVA [32]. In regard to the number of attempts, Tran and colleagues [11] found a positive significant association in favor of US-guided method in adults, although with several discrepancies.

The variability in the estimates could be a consequence of the differences in both the way to measure the experience and training of operators and the device used [10, 25]. In the same line, providers have extensive experience in the traditional technique, which could underestimate the potential benefit of the US-guided technique [33]. On the other hand, there is neither agreement on the groups studied nor on the distribution by age group (< 3, < 7, < 10 and/or < 18 years).

Regarding efficiency, no studies found a significant positive effect of US versus traditional method in the time spent in SPC insertion, as stated by a recent RCT which has not been included in any systematic review [34]. The lack of significant findings may result from the large variability among studies. Several methodology-related reasons could be behind this variability, such as differences in the definitions of procedure length [35–37]. Likewise, history of DVA has not been well-defined in all studies. Since a validated scale for reporting DVA is not available, each study could define it in a different way [12]. Thus, the use of a predicting scale to recognize patients with DVA may improve the methodological quality of further studies [38, 39]. The use of validated methods is important because the inexperience of the operator could be mistaken for a difficult access, which involves a risk of bias [25].

Regarding patients’ satisfaction, although our findings only referred to adult population, the higher rates were obtained when US-guided method was used, except in one study [11, 12, 24]. One reason which could respond to the variability in findings about satisfaction, and which is applicable to the rest of outcomes, is the context where studies were developed. It is logical to think that both the pressure on healthcare personnel and the target response time vary among hospital departments, [40] so the SPC insertion technique is not carried out on equal terms. For example, in the treatment of cardiac arrests in pediatric population the European Resuscitation Council Guidelines 2021 recommend getting a vascular access in 5 min at most and suggest the use of US to guide cannulation in competent providers [41]. Considering that ED is a hospital area where cardiac arrests are common, [42] it seems reasonable to assume that there might be differences between different hospital areas in the US-guided method for SPC cannulation.

In spite of the possible controversial findings and the wide heterogeneity in the studies included, US-guided method seems to be a useful technique that could improve the quality indexes regarding the care and safety of the patient. Likewise, these findings might help to reduce the central venous catheterization (CVC) in patients with DVA, as stated by Au and colleagues, [43] which could avoid the CVC-associated complications [44]. However, this technique requires an in-depth knowledge by operators, [26, 45] and the traditional technique training is more established compared to the US-guided method, [33] although the US training could be further enhanced by the use of long peripheral catheters in DVA patients [46, 47]. The development of training programs could enhance the use of this alternative and its benefits [48]. Furthermore, the inexistence of significant benefit in the time spent in US-guided method, together with pressure on healthcare personnel in certain hospital departments and the availability of the required US equipment, could limit the use of this alternative. However, according to ERPIUP Consensus, those US-guided SPC used to canalize deep vein could have a duration of about 24 h or less [7], so it is necessary further research in order to elucidate the differences between short and long peripheral catheters in terms of feasibility and duration. Likewise, operators should consider this statement in the different clinical situations because the US-guided SPC insertion could be a great tool in the immediate treatment of DVA patients, but it could also be a handicap in long-term intravenous treatments, particularly if the canalized vein is deep.

### Limitations of the study

Firstly, there are methodological differences between the studies included, especially in the quantitative synthesis and the operationalization of variables. Secondly, there is a large overlap between previous systematic reviews included in our umbrella review. (Table S8). On the other hand, the ‘peripheral intravenous catheters (PIVCs)’ terminology is ambiguous, and its use in some of the included previous studies could be associated with the existence of selection bias specially if some of them have included LPC or MC within the PIVCs. Finally, considering that several systematic reviews included compared data from both RCT and observational studies, the findings must be cautiously interpreted because the analysis of effectiveness of clinical techniques through observational studies could lead to several limitations. These limitations could be counterbalanced with further RCTs that include patients with similar age categorized by hospital areas, using a standardized definition of both the PVADs and the procedure length and a validated scale for DVA definition. Likewise, the comparison between both techniques should be carried out through operators who report similar levels of expertise.

## Conclusions

US-guided method for SPC insertions has shown itself to be more effective in terms of success rates and patient satisfaction when compared to traditional landmark method in adult and pediatric population. Our results have clinical importance because it could benefit patients with special conditions which difficult the venous access or whose venous resources have particular characteristics. Likewise, this method, by reducing the common technique-associated complications, could increase the quality of healthcare and stablish an optimal level of compliance between patients and providers. With a moderate certainty level, the US-guided method is postulated as a valid alternative in comparison with the traditional method in the SPC insertion. A higher success rate, a lower number of unsuccessful attempts and a lower presence of associated complications make the US-guided method the main option in patients with DVA.

## Supplementary Information

**Additional file 1. Table S1**. PRISMA 2020 Checklist.** Table S2**. Search strategy from database inception to 23rd February 2022.** Table S3**. Excluded studies from the umbrella review with reasons.** Table S4**. Methodological quality assessment by AMSTAR-2 (a critical appraisal tool for systematic reviews of healthcare interventions). **Table S5**. Certainty of evidence according to Grading of Recommendation Assessment, Development, and Evaluation (GRADE).** Table S6**. Evidence map.** Table S7**. Characteristics of the randomized controlled trials which compare traditional versus US-guided method for SPC insertion and have been included in the previous meta-analyses.** Table S8**. Study overlaps in included systematic reviews and meta-analyses.

## Data Availability

All data analyzed during this study are included in this article [and its supplementary information files].

## Abbreviations

AMSTAR: Assessment of multiple systematic reviews
CI: Confidence interval
CVC: Central venous catheterization
DVA: Difficult venous access
ED: Emergency department
EMBASE: Excerpta Medica Data Base
ES: Effect size
GRADE: Grading of Recommendations, Assessment, Development, and Evaluation
I^2^: Heterogeneity
ICU: Intensive unit care
LPC: Long peripheral catheters
MC: Midline catheters
MEDLINE: Medical Literature Analysis and Retrieval System Online
MD: Mean difference
NA: Not available
No: Number
OR: Odds ratio
OR*: operating room
PICC: Peripherally Inserted Central Catheter
PICO: Patient Intervention Comparison And Outcome
PIVCs: Peripheral intravenous catheters
PRIOR: Preferred Reporting Items for Overviews of Reviews
PRISMA: Preferred Reporting Items for Systematic Review and Meta-Analyses
PROSPERO: International Prospective Register of Systematic Reviews
PVADs: Peripheral venous access devices
RCT: Randomized controlled trial
RR: Relative Risk
SMD: Standardized mean difference
SPC: Short peripheral catheter
STATA: Statistics and data
US: Ultrasonography
WMD: Weighted mean difference
WOS: Web of science
