# Supplementary material for: Ultrasound-guided versus traditional method for peripheral venous access: an umbrella review
Source: BMC Nurs. 2022 Nov 9;21:307. doi: 10.1186/s12912-022-01077-9 (PMC9644458; doi:10.1186/s12912-022-01077-9)
Supplement: Supplementary file 1 — Additional file 1. Table S1. PRISMA 2020 Checklist. Table S2. Search strategy from database inception to 23rd February 2022. Table S3. Excluded studies from the umbrella review with reasons. Table S4. Methodological quality assessment by AMSTAR-2 (a critical appraisal tool for systematic reviews of healthcare interventions). Table S5. Certainty of evidence according to Grading of Recommendation Assessment, Development, and Evaluation (GRADE). Table S6. Evidence map. Table S7. Characteristics of the randomized controlled trials which compare traditional versus US-guided method for SPC insertion and have been included in the previous meta-analyses. Table S8. Study overlaps in included systematic reviews and meta-analyses. [file 12912_2022_1077_MOESM1_ESM.docx]

**Table S1. PRISMA 2020 Checklist.**

| **Section and Topic** | **Item #** | **Checklist item** | **Page(s) where item is reported** |
| --- | --- | --- | --- |
| **TITLE** | | |  |
| Title | 1 | Identify the report as a systematic review. | 1 |
| **ABSTRACT** | | |  |
| Abstract | 2 | See the PRISMA 2020 for Abstracts checklist. | 2,3 |
| **INTRODUCTION** | | |  |
| Rationale | 3 | Describe the rationale for the review in the context of existing knowledge. | 4,5 |
| Objectives | 4 | Provide an explicit statement of the objective(s) or question(s) the review addresses. | 5 |
| **METHODS** | | |  |
| Eligibility criteria | 5 | Specify the inclusion and exclusion criteria for the review and how studies were grouped for the syntheses. | 6,7 |
| Information sources | 6 | Specify all databases, registers, websites, organisations, reference lists and other sources searched or consulted to identify studies. Specify the date when each source was last searched or consulted. | 6 |
| Search strategy | 7 | Present the full search strategies for all databases, registers and websites, including any filters and limits used. | Supplementary material: 5 |
| Selection process | 8 | Specify the methods used to decide whether a study met the inclusion criteria of the review, including how many reviewers screened each record and each report retrieved, whether they worked independently, and if applicable, details of automation tools used in the process. | 6,7,8 |
| Data collection process | 9 | Specify the methods used to collect data from reports, including how many reviewers collected data from each report, whether they worked independently, any processes for obtaining or confirming data from study investigators, and if applicable, details of automation tools used in the process. | 7,8 |
| Data items | 10a | List and define all outcomes for which data were sought. Specify whether all results that were compatible with each outcome domain in each study were sought (e.g. for all measures, time points, analyses), and if not, the methods used to decide which results to collect. | 7 |
|  | 10b | List and define all other variables for which data were sought (e.g. participant and intervention characteristics, funding sources). Describe any assumptions made about any missing or unclear information. | 7 |
| Study risk of bias assessment | 11 | Specify the methods used to assess risk of bias in the included studies, including details of the tool(s) used, how many reviewers assessed each study and whether they worked independently, and if applicable, details of automation tools used in the process. | 7,8 |
| Effect measures | 12 | Specify for each outcome the effect measure(s) (e.g. risk ratio, mean difference) used in the synthesis or presentation of results. | 9 |
| Synthesis methods | 13a | Describe the processes used to decide which studies were eligible for each synthesis (e.g. tabulating the study intervention characteristics and comparing against the planned groups for each synthesis (item #5)). | 9 |
|  | 13b | Describe any methods required to prepare the data for presentation or synthesis, such as handling of missing summary statistics, or data conversions. | 9 |
|  | 13c | Describe any methods used to tabulate or visually display results of individual studies and syntheses. | 9 |
|  | 13d | Describe any methods used to synthesize results and provide a rationale for the choice(s). If meta-analysis was performed, describe the model(s), method(s) to identify the presence and extent of statistical heterogeneity, and software package(s) used. | 9 (Not meta-analysis) |
|  | 13e | Describe any methods used to explore possible causes of heterogeneity among study results (e.g. subgroup analysis, meta-regression). | Not meta-analysis |
|  | 13f | Describe any sensitivity analyses conducted to assess robustness of the synthesized results. | Not meta-analysis |
| Reporting bias assessment | 14 | Describe any methods used to assess risk of bias due to missing results in a synthesis (arising from reporting biases). | 7,8 |
| Certainty assessment | 15 | Describe any methods used to assess certainty (or confidence) in the body of evidence for an outcome. | 8 |
| **RESULTS** | | |  |
| Study selection | 16a | Describe the results of the search and selection process, from the number of records identified in the search to the number of studies included in the review, ideally using a flow diagram. | 10, 11 |
|  | 16b | Cite studies that might appear to meet the inclusion criteria, but which were excluded, and explain why they were excluded. | Supplementary material: 6-16 |
| Study characteristics | 17 | Cite each included study and present its characteristics. | 10  Table 1 |
| Risk of bias in studies | 18 | Present assessments of risk of bias for each included study. | 11, 12 |
| Results of individual studies | 19 | For all outcomes, present, for each study: (a) summary statistics for each group (where appropriate) and (b) an effect estimate and its precision (e.g. confidence/credible interval), ideally using structured tables or plots. | 12-15 |
| Results of syntheses | 20a | For each synthesis, briefly summarise the characteristics and risk of bias among contributing studies. | 12-15 |
|  | 20b | Present results of all statistical syntheses conducted. If meta-analysis was done, present for each the summary estimate and its precision (e.g. confidence/credible interval) and measures of statistical heterogeneity. If comparing groups, describe the direction of the effect. | 12-15 (Not meta-analysis) |
|  | 20c | Present results of all investigations of possible causes of heterogeneity among study results. | 16-18 (Not meta-analysis) |
|  | 20d | Present results of all sensitivity analyses conducted to assess the robustness of the synthesized results. | Not meta-analysis |
| Reporting biases | 21 | Present assessments of risk of bias due to missing results (arising from reporting biases) for each synthesis assessed. | 11,12 |
| Certainty of evidence | 22 | Present assessments of certainty (or confidence) in the body of evidence for each outcome assessed. | 12 |
| **DISCUSSION** | | |  |
| Discussion | 23a | Provide a general interpretation of the results in the context of other evidence. | 16 |
|  | 23b | Discuss any limitations of the evidence included in the review. | 18,19 |
|  | 23c | Discuss any limitations of the review processes used. | 18,19 |
|  | 23d | Discuss implications of the results for practice, policy, and future research. | 19 |
| **OTHER INFORMATION** | | |  |
| Registration and protocol | 24a | Provide registration information for the review, including register name and registration number, or state that the review was not registered. | 6 |
|  | 24b | Indicate where the review protocol can be accessed, or state that a protocol was not prepared. | 6 |
|  | 24c | Describe and explain any amendments to information provided at registration or in the protocol. | 8 |
| Support | 25 | Describe sources of financial or non-financial support for the review, and the role of the funders or sponsors in the review. | 20 |
| Competing interests | 26 | Declare any competing interests of review authors. | 20 |
| Availability of data, code and other materials | 27 | Report which of the following are publicly available and where they can be found: template data collection forms; data extracted from included studies; data used for all analyses; analytic code; any other materials used in the review. | Table 1 and Supplementary material: data extracted, search strategy, excluded studies, AMSTAR-2 tool, GRADE tool, evidence map. |

*From: Page MJ, MacKenzie JE,Bossuyt PM, et al. The PRISMA 2020 statement: an updated guideline for reporting systematic reviews. BMJ 2021;372. More information at* [*https://www.bmj.com/content/372/bmj.n71*](https://www.bmj.com/content/372/bmj.n71)

**Table S2. Search strategy from database inception to 23^rd^ February 2022.**

| **Database** | **Search term** | **Results** |
| --- | --- | --- |
| MEDLINE (via PubMed) | (Ultrasound OR Echoguided OR Ultrasound-guided OR Sonography OR Ultrasonography OR Echo) AND (‘Peripheral vein’ OR ‘Peripheral venous’ OR ‘Peripheral intravenous’ OR Vein OR Venous OR Intravenous OR Vascular) AND (Cannulation OR Access OR Catheterization) NOT (Arterial OR Radial OR Femoral OR ‘PICC’ OR Central OR Midline) AND (Review OR 'systematic review' OR 'systematic literature review' OR Meta OR 'meta-analysis' OR 'meta-analyses' OR 'meta analysis' OR ‘meta analyses’) | 1566 |
| EMBASE (via Scopus) | ("Ultrasound" OR "Echo") AND ("venous" OR "Vein") AND ("Catheterization") AND ("Review" OR "Meta" OR "systematic review" OR "systematic literature review" OR "meta-analysis" OR "meta-analyses" OR "meta analysis" OR "meta analyses") | 767 |
| Web of Science | (Ultrasound OR echoguide OR Sonography OR Echo) AND (Vein OR Venous OR Vascular) AND (Access OR Catheterization) NOT (Arterial OR Radial OR Femoral OR ‘PICC’ OR Central OR Midline) AND (Review OR 'systematic review' OR 'systematic literature review' OR Meta OR 'meta-analysis' OR 'meta-analyses' OR 'meta analysis' OR ‘meta analyses’) | 609 |
| Cochrane Library | (Ultrasound OR Echoguided OR Ultrasound-guided OR Sonography OR Ultrasonography OR Echo) AND (‘Peripheral vein’ OR ‘Peripheral venous’ OR ‘Peripheral intravenous’ OR Vein OR Venous OR Intravenous OR Vascular) AND (Cannulation OR Access OR Catheterization) NOT (Arterial OR Radial OR Femoral OR ‘PICC’ OR Central OR Midline) AND ('systematic review' OR 'systematic literature review' OR 'meta-analysis' OR 'meta-analyses' OR 'meta analysis' OR 'meta analyses') in Title Abstract Keyword – in Cochrane Reviews | 4 |
| **TOTAL** |  | 2946 |

**Table S3. Excluded studies from the umbrella review with reasons.**

| **Exclusion reasons** | **Reference** |
| --- | --- |
| Study design  (n=88) | 1. Terkawi A, Karakitsos D, Elbarbary M, et al. Ultrasound for the anesthesiologists: present and future. The Scientific World Journal 2013;2013:683685. 2. Reusz G, Csomos A. Peripheral venous access with ultrasound guidance. Orvosi Hetilap 2013; 154:187-190. 3. Levy J, Bachur R. Bedside ultrasound in the pediatric emergency department. Current Opinion in Pediatrics 2008;20:235-242. 4. Gallieni M, Pittiruti M, Biffi R. Vascular Access in oncology patients. CA Cancer J Clin 2008; 58:323-346. 5. Merrer J, Lefrant J, Timsit J. How to improve central venous catheter use in intensive care unit? Annales Francaises d’Anesthesie et de Reanimation 2006;25:180-188. 6. Dhamija E, Thulkar S, Bhatnagar S. Utility and potential of bedside ultrasound in palliative care. Indian J Palliat Care 2015; 21:132-136. 7. Vezzani A, Manca T, Vercelli A, Braghieri A, Magnacavallo A. Ultrasonography as a guide during vascular access procedures and it the diagnosis of complications. J Ultrasound 2013; 16:161-170. 8. Su E, Dalesio N, Pustavoitau, A. Point-of-care ultrasound in pediatric anesthesiology and critical care medicine. Can J Anesth 2018; 65:485-498. 9. Weiner M, Geldard P, Mittnacht A. Ultrasound-guided vascular access: a comprehensive review. Journal of Cardiothoracic and vascular anesthesia 2013;27:345-360. 10. Munshey F, Parra DA, MacDonnel C, Matava C. Ultrasound-guided techniques for peripheral intravenous placement in children with difficult venous access. Pediatric Anesthesia 2020;30:108–115. 11. Vargas S, Carrillo A. Use and training in ultrasound in the emergency room. Universitas Medica 2013;54:353-360. 12. Detaille T, Pirotte T, Veyckemans F. Vascular access in the neonate. Best Pract Res Clin Anaesthesiol 2010;24:403-418. 13. Yamamoto T, Schindler E. Notfälle im Rettungsdienst und in der Klinik: Gefäßzugänge bei Kindern. Anasthesiologie, Intensivmedizin, Notfallmedizin, Schmerztherapie 2017; 52: 55-64. 14. Yamagami Y, Ueki S, Matoba K, Makimoto K. Effectiveness of ultrasound-guided peripheral intravenous cannulation in pediatric patients aged under three years: a systematic review protocol. JBI Database System Rev Implement Rep 2018; 1635–38. 15. Bennett J, Cheung M. Intravenous access in children. Paediatrics and child health 2020;30:224-229. 16. Deshpande R, Akhtar S, Haddadin A. Utility of ultrasound in the ICU. Current opinion in anaesthesiology 2014;27:123-132. 17. Corcoran F, Bystrzycki A, Masud S, Mazur SM, Wise D, Harris T. Ultrasound in pre-hospital trauma care. Trauma 2016;18:101-110. 18. Maecken T, Grau T. Ultrasound imaging in vascular access. Crit Care Med 2007;35:S178-S185. 19. Delacruz N, Malia L, Dessie A. Point-of-Care Ultrasound for the evaluation and management of febrile infants. Pediatric emergency care 2021;37:e886-e892. 20. Rippey J, Royse A. Ultrasound in trauma. Best practice & research. Clinical anaesthesiology 2009;23:343-362. 21. Haas N. Clinical review: vascular access for fluid infusion in children. Critical Care 2004;8:478-484. 22. Donaldson J. Pediatric vascular access. Pediatric Radiology 2006;36:386-397. 23. Naik V, Mantha S, Rayani B. Vascular access in children. Indian journal of anaesthesia 2019;63:737-745. 24. Sahlani L, Thompson L, Vira A, Panchal AR. Bedside ultrasound procedures: musculoskeletal and non-musculoskeletal. European Journal of Trauma and Emergency Surgery 2016;42:127-138. 25. Errando C. Ultrasound images for venous puncture and venous access. Revista Española de Anestesiología y Reanimación 2008;55:593-594. 26. Haroon-Mowahed Y, Cheen Ng S, Barnett S, West S. Ultrasound in paediatric anaesthesia – A comprehensive review. Ultrasound (Leeds, England) 2021;29:112-122. 27. Guillory R, Gunter O. Ultrasound in the surgical intensive care unit. Current opinion in critical care 2008;14:415-422. 28. O’Brien A, Brady R. Point-of-care ultrasound in paediatric emergency medicine. Journal of paediatrics and child health 2016;52:174-180. 29. Andruszkiewicz P, Sobczyk D. Ultrasound in critical care. Anaesthesiology intensive therapy 2013;45:177-181. 30. Le Coz J, Orlandini S, Titomanlio L, Rinaldi, VE. Point of care ultrasonography in the pediatric emergency department. Italian journal of pediatrics 2018:44:87 31. Dietrich A, Coley B. Bedside pediatric emergency evaluation through ultrasonography. Pediatric radiology 2008;38 Suppl 4:S679-84. 32. Danski MTR, Oliveira AMD, Meier MJ, Pedrolo E. Effectiveness of ultrasonography-guided peripheral venous access: an integrative review. Enfermería Global 2016;15:354-367. 33. Blanco P. Ultrasound-guided peripheral venous cannulation in critically ill patients: a practical guideline. Ultrasound Journal 2019;11:27. 34. Johnson GGRJ, Kirkpatrick AW, Gillman LM. Ultrasound in the surgical ICU: uses, abuses, and pitfalls. Current opinion in critical care 2019;25:675-687. 35. Denault A, Lamarche Y, Rochon A, et al. Innovative approaches in the perioperative care of the cardiac surgical patient in the operating room and intensive care unit. The Canadian journal of cardiology 2014;30:S459-77. 36. Maracaja L, Blitz D, Maracaja DLV, Walker C, Freudzon, L. One Hand and One Eyesight Real-Time Ultrasound Guidance: The Use of CAD Design and 3D Printing for Development of New Tools to Improve Vascular Access. Journal of cardiothoracic and vascular anesthesia 2020;34:2136-2139. 37. Murphy E, Ross R, Jones R, et al. Imaging in vascular access. Cardiovascular Engineering and Technology 2017;8:255-272. 38. Gupta PK, Gupta K, Dwivedi AND, Jain M. Potential role of ultrasound in anesthesia and intensive care 2011;5:11-19. 39. Gleeson T, Blehar D. Point-of-Care Ultrasound in Trauma. Seminars in ultrasound, CT, and MR 2018;39:374-383. 40. Vakamudi M, Ravulapalli H, Karthikeyan R. Recent advances in paediatric cardiac anaesthesia. Indian journal of anaesthesia 2012;56:485-490. 41. Naji A, Chappidi M, Ahmed A, Monga A, Sanders J. Perioperative Point-of-Care Ultrasound Use by Anesthesiologists. Cureus 2021;13:e15217. 42. White A, Lopez F, Stone P. Developing and Sustaining an Ultrasound-Guided Peripheral Intravenous Access Program for Emergency Nurses. Advanced Emergency Nursing Journal 2010;32:173-188. 43. Braverman J. Bedside ultrasound for procedural assistance in pediatrics. Pediatric annals 2021;50:e404-e410. 44. Shahzad A, Saad MNM, Walter N, Malik AS, Meriaudeau F. A review of subcutaneous veins localization using imaging techniques. Current Medical Imaging Reviewa 2014;10:125-133. 45. Kayashima K, Uezono S, Andropoulos D. Chapter 10: Vascular acces and monitoring. In: Andropoulos DB, Stayer S, Mossad EB, Miller-Hance WC, eds. Anesthesia for Congenital Heart Disease: Third Edition. USA: John Wiley & Sons, Inc, 2015;199-229. 46. Holm HH, Skjoldbye B. Interventional ultrasound. Ultrasound in Medicine and Biology 1996;22:773789. 47. Church J, Jarboe M. Vascular access in the pediatric population. Surgical Clinics of North America 2017;97:113-128. 48. Pirotte T. Ultrasound-guided vascular access in adult and children: beyond the internal jugular vein puncture. Acta Anaesthesiologica Belgica 2008;59:157-166. 49. Barrett A, Squire R. Vascular access. In: Stringer M, Oldham K, Mouriquand P, eds. Pediatric Surgery and Urology: Long-Term Outcomes, Second Edition. Cambridge: Cambridge University Press, 2006; 947-957. 50. Cheung E, Baerlocher MO, Asch M, Myers A. Venous access: A practical review for 2009. Canadian Family Physician 2009;55:494-496. 51. Morrow D, Cupp J, Schrift D, Nathanson R, Soni NJ. Point-of-Care Ultrasound in established settings. Southern Medical Journal 2018;111:373-381. 52. Schmidt G, Blaivas M, Conrad S, et al. Ultrasound-guided vascular access in critical illness. Intensive Care Medicine 2019;434-446. 53. Schwemmer U, Brederlau J. Ultrasound methods in anesthesiology: vascular puncture under sonography. Anasthesiologie Intensivmedizin Notfallmedizin Schmerztherapie 2006;41:740-748. 54. Lewis GC, Crapo SA, Williams JG. Critical skills and procedures in emergency medicine: vascular access skills and procedures. Emergency Medicine Clinics of North America 2013;31:59-86. 55. Shaahinfar A, Ghazi-Askar ZM. Procedural Applications of Point-of-Care Ultrasound in Pediatric Emergency Medicine. Emergency Medicine Clinics of North America 2021;39:529-554. 56. Mayordomo-Colunga J, González-Cortés R, Bravo MC. Point-of-care ultrasound: Is it time to include it in the paediatric specialist training program? Anales de Pediatría 2019;91:206.e1-206.e13. 57. Lee SY, Frankel HL. Ultrasound and other imaging technologies in the intensive care unit. Surgical Clinics of North America 2000;80:975-1003. 58. Almeida C. Vascular access: the impact of ultrasonography. Einstein (Sao Paulo) 2016;14:561-566. 59. Tirado A, Nagdev A, Henningsen C, Breckon P, Chiles K. Ultrasound-guided procedures in the emergency department-needle guidance and localization. Emergency Medicine Clinics of North America 2013;31:87-115. 60. Millington SJ, Hendin A, Shiloh AL, Koenig S. Better with ultrasound: peripheral intravenous catheter insertion. Chest 2020;157:369-375. 61. Galvan D, Matsushima K, Frankel H. Ultrasound in the surgical intensive care unit. Israel Medical Association Journal 2011;13:566-567. 62. Lin S. Procedural ultrasound in pediatric patients: techniques and tips for accuracy and safety. Pediatric emergency medicine practice 2016;13:1-5. 63. Seddon D, McLeod M. Equipment and monitoring in paediatric anaesthesia. Anaesthesia and Intensive Care Medicine 2018;19:409-413. 64. Leslie D, Froom S, Gildersleve C. Equipment and monitoring for paediatric anaesthesia. Anaesthesia and Intensive Care Medicine 2015;16:389-394. 65. Fraga MV, Stoller JZ, Glau CL, et al. Seeing is believing: ultrasound in pediatric procedural performance. Pediatrics 2019;144:e20191401. 66. Nemcek A. Use of ultrasonography in vascular interventions. Seminars in Interventional Radiology 1997;14:455-469. 67. Aygun M, Yaman HE, Bayindir A. The use of Ultrasonography-guided peripheral intravenous access in Emergency Department Patients with Difficult Venous Access. Eurasian Journal of Emergency Medicine 2010;9:9-16. 68. Abo A, Kelley K, Kuppermann N, Cusick S. Ultrasound equipment for the pediatric emergency department: a review. Pediatric Emergency Care 2011;27:220-229. 69. Jain P, Ranganathan P. Ultrasound in anaesthesia. Indian Journal of Anaesthesia 2007;51:176-183. 70. Schindler E, Schears GJ, Hall SR, Yamamoto T. Ultrasound for vascular access in pediatric patients. Paediatric Anaesthesia 2012;22:1002-1007. 71. Mbamalu D, Banerjee A. Methods of obtaining peripheral venous access in difficult situations. Postgraduate Medical Journal 1999;75:459-462. 72. Blanco P. Ultrasound-guided vascular cannulation in critical care patients: A practical review. Medicina Intensiva 2016;40:560-571. 73. Rahman O, Willis L. Vascular procedures in the critically ill obese patient. Critical Care Clinics 2010;26:647-660. 74. Shandu N. Ultrasound imaging in anesthesia: an overview of vascular access and peripheral nerve blocks. Seminars in Anesthesia, Perioperative Medicine and Pain 2007;26:197-209. 75. Gottlieb M, Sundaram T, Holladay D, Nakitende D. Ultrasound-guided peripheral intravenous line placement: A narrative review of evidence-based best practices. Western Journal of Emergency Medicine 2017;18:1047-1054. 76. Reusz G, Csomos A. The role of ultrasound guidance for vascular access. Current opinion in Anaesthesiology 2015;28:710-716. 77. Koster G, Van Der Horst I. Critical care ultrasonography in circulatory shock. Current Opinion in Critical Care 2017;23:326-333. 78. Moore C. Ultrasound-guided procedures in emergency medicine. Ultrasound Clinics 2011;6:277-289. 79. Bortcosh W, Shaahinfar A, Sojar S, Klig JE. New directions in point-of-care ultrasound at the crossroads of paediatric emergency and critical care. Current Opinion in Pediatrics 2018;30:350-358. 80. Lamperti M, Bodenham A, Pittiruti M, et al. International evidence-based recommendations on ultrasound-guided vascular access. Intensive Care Medicine 2012;38:1105-1117. 81. Barr L, Hatch N, Roque PJ, Wu TS. Basic ultrasound-guided procedures. Critical Care Clinics 2014;30:275-304. 82. Miller L, Stoller J, Fraga M. Point-of-care ultrasound in the neonatal ICU. Current opinion in pediatrics 2020;32:216-227. 83. Yıldızdaş D, Aslan N. A new stethoscope for pediatric intensivists: Point-of-care ultrasound. Turk Pediatri Arsivi 2020;55:345-353. 84. Leung S. A review of pediatric ultrasound-guided peripheral intravenous access. Clinical Pediatric Emergency Medicine 2015;16:240-243. 85. Breakey N, Osterwalder J, Mathis G, Lehmann B, Sauter TC. Point of care ultrasound for rapid assessment and treatment of palliative care patients in acute medical settings. European journal of internal medicine 2020;81:7-14. 86. Moore C. Ultrasound first, second, and last for vascular access. Journal of Ultrasound in Medicine 2014;33:1135-1142. 87. Nakayama Y, Takeshita J, Nakajima, Y Shime N. Ultrasound-guided peripheral vascular catheterization in pediatric patients: a narrative review. Critical Care 2020;24:592. 88. Khan MS, Sabnis VB, Phansalkar DS, Prasad SP, Karnam AHF. Use of ultrasound in peripheral venous catheterization in adult emergency and critical care units. Anaesthesia, Pain and Intensive Care 2015;19:303-310. |
| Not method comparison of interest  (n=14) | 1. Chen J, Wang L, Lin Y. Comparing short-, long-, and oblique-axis approaches to ultrasound-guided internal jugular venous catheterization: A meta-analysis of randomized controlled trials. Journal of Trauma and Acute Care Surgery 2019;86:516-523. 2. Gerlando F, Scaccaglia D, Artioli G, Sarli L, Romano R. Intraosseus access vs ecoguided peripherical venous access in emergency and urgency: a systematic review. Acta bio-medica: Atenei Parmensis 2021;92:e2021334 3. Maitra S, Bhattacharjee S, Baidya D. Comparison of long-, short-, and oblique-axis approaches for ultrasound-guided internal jugular vein cannulation: A network meta-analysis. Journal of Vascular Access 2020;21:204-209. 4. Qin KR, Ensor N, Barnes R, Englin A, Nataraja RM, Pacilli M. Long peripheral catheters for intravenous access in adults and children: A systematic review of the literature. The journal of vascular access 2021;22:767-777. 5. Miao S, Wang X, Zou L, et al. Safety and efficacy of the oblique-axis plane in ultrasound-guided internal jugular vein puncture: a meta-analysis. Journal of International Medical Research 2018;46:2587-2594. 6. Schnabel A, Meyer-Frießem CH, Zahn PK, Pogatzki-Zahn EM. Ultrasound compared with nerve stimulation guidance for peripheral nerve catheter placement: a meta-analysis of randomized controlled trials. British Journal of Anaesthesia 2013;111:564-572. 7. Heinrichs J, Fritze Z, Klassen T, Curtis S. A systematic review and meta-analysis of new interventions for peripheral intravenous cannulation of children. Pediatric Emergency Care 2013;29:858-866. 8. Gao YB, Yan JH, Ma JM, et al. Effects of long axis in-plane vs short axis out-of-plane techniques during ultrasound-guided vascular access. American Journal of Emergency Medicine 2016;34:778-783. 9. Liu C, Mao Z, Kang H et al. Comparison between the long-axis/in-plane and short-axis/out-of-plane approaches for ultrasound-guided vascular catheterization: An updated meta-analysis and trial sequential analysis. Ther Clin Risk Manag 2018;14:331-340. 10. Yen K, Gorelick M. New biomedical devices that use near-infrared technology to assist with phlebotomy and vascular access. Pediatr Emerg Care 2013; 29:383-385. 11. Badger J. Long peripheral catheters for deep arm vein venous access: a systematic review of complications. Heart and lung 2019;48:222-225. 12. Shi J, Shen J, Xiang Z, Liu X, Lu, T, Tao X. Dynamic needle tip positioning versus palpation and ultrasound for arteriovenous puncture: a meta-analysis. Ultrasound in medicine and biology 2021;47:2233-2242. 13. Wu S, Huang J, Jiang Z et al. Internal jugular vein versus subclavian vein as the percutaneous insertion site for totally implantable venous access devices: a meta-analysis of comparative studies. BMC Cancer 2016;16:747. 14. Atti V, Turagam M, Garg J, et al. Subclavian and Axillary Vein Access Versus Cephalic Vein Cutdown for Cardiac Implantable Electronic Device Implantation: A Meta-Analysis. JACC. Clinical electrophysiology 2020;6:661-671. |
| Not including peripheral venous access  (n=25) | 1. Keenan S. Use of ultrasound to place central lines. Journal of Critical Care 2002;17:126-137. 2. Randolph AG, Cook DJ, Gonzales CA, Pribble CG. Ultrasound guidandce for placement of central venous catheters: a meta-analysis of the literature. Critical Care Medicine 1996;24:2053-2058. 3. Wang T, Wang M, Martin A. Meta-analysis of ultrasound-guided versus conventional vascular access for electrophysiology procedures and catheter ablation. J Am Coll Cardiol 2019;73:374. 4. De Souza TH, Brandão MB, Nadal JAH, Nogueira RJN. Ultrasound Guidance for Pediatric Central Venous Catheterization: A Meta-analysis. Pediatrics 2018;142:e20181719. 5. Gurien L, Blakely M, Crandall et al. Meta-analysis of surgeon-performed central line placement: real-time ultrasound versus landmark technique. Journal of Trauma and Acute Care Surgery 2018;84:655-663. 6. Brass P, Hellmich M, Kolodziej L, Schick G, Smith A. Ultrasound guidance versus anatomical landmarks for internal jugular vein catheterization. Cochrane Database of Systematic Reviews 2015; 1:CD006962. 7. Li QL, Yan MQ, Zhang XJ, Lu ZQ, Lin C. Influence of PICC ultrasound guidance on elbow puncture and catheterization and its complications: A systematic review. Chinese Journal of Evidence-Based Medicine 2013;13:816-826. 8. Wu S.-Y, Ling Q, Cao L-H, Wang J, Xu M-X, Zeng WA. Real-time two-dimensional ultrasound guidance for central venous cannulation: a meta-analysis. Anesthesiology 2013; 118:361-375. 9. Jiang L, Zhang M, Ma Y, Lalu MM, Barron CC, Fergusson DA. Ultrasound-guided subclavian vein catheterization: a systematic review and meta-analysis: several facts need to be noticed. Crit Care Med 2015; 43: e474-e475 10. Xiong X, Xiong Y, Liu G. Systematic review and meta-analysis: safety of ultrasound-guided peripheral venipuncture and catheterization. Ann Palliat Med 2021;10(11):11721-11732. 11. Patil V, Jaggar S. Ultrasound guided internal jugular vein Access in children and infant: A meta-analysis. Paediatric Anaesthesia 2010;20:474-475. 12. Lalu M, Fayad A, Ahmed O, et al. Ultrasound-guided subclavian vein catheterization: a systematic review and meta-analysis. Crit Care Med 2015;43:1498-1507. 13. Hind D, Calvert N, McWilliams et al. Ultrasonic locating devices for central venous cannulation: meta-analysis. BMJ 2003;327:361-364. 14. Attie G, Flumignan C, Silva M, et al. What do cochrane systematic reviews say about ultrasound-guided vascular access? Sao Paulo Medical Journal 2019;137:284-291. 15. Sobolev M, Shiloh AL, Di Biase L, Slovut DP. Ultrasound-guided cannulation of the femoral vein in electrophysiological procedures: a systematic review and meta-analysis. Europace 2017;19:850-855. 16. Sigaut S, Skhiri A, Stany I, et al. Ultrasound guided internal jugular vein Access in children and infant: a meta-analysis of published studies. Paediatric Anaesthesia 2009;19:1199-1206. 17. Lau C, Chamberlain R. Ultrasound-guided central venous catheter placement increases success rates in pediatric patients: a meta-analysis. Pediatric Research 2016; 80:178-184. 18. Li Z, Chen L. Comparison of ultrasound-guided modified Seldinger technique versus blind puncture for peripherally inserted central catheter. A meta-analysis of randomized controlled trials. Critical Care 2015;19:64. 19. Mehta N, Valesky WW, Guy A, Sinert R. Systematic review: Is real-time ultrasonic-guided central line placement by ED physicians more successful than the traditional landmark approach? Emergency Medicine Journal 2013; 30:355-359. 20. Calvert N, Hind D, McWilliams R, Thomas SM, Beverley C, Davidson A. The effectiveness and cost-effectiveness of ultrasound locating devices for central venous access: A systematic review and economic evaluation. Health Technology Assessment 2003;7:1-84. 21. Shime N, Hosokawa K, MacLaren G. Ultrasound imaging reduces failure rates of percutaneous central venous catheterization in children. Pediatric Critical Care Medicine 2015;16:718-725. 22. Asbeutah AAA, Asbeutah AM, Ibebuogu UN, Khouzam RN. Meta-Analysis of Outcomes in Ultrasound Guided Versus Traditional Guided Vascular Access for Interventional Cardiac and Peripheral Vascular Procedures. American Journal of Cariology 2021;148:176-178. 23. Chi DM, Yu H, Lan HD, Liu B. Effectiveness and safety of the ultrasound guidance for internal jugular vein catheterization in pediatric patients: a meta-analysis. Chinese Journal of Evidence-Based Medicine 2016;16:1465-1471. 24. Zhang Q, Ma HP, Li X, Wu JJ, Wang J, Zheng H. Safety and effectiveness of real time two dimensional ultrasound guidance for internal jugular vein cannulation: A systematic review. Chinese Journal of Evidence-Based Medicine 2015;15:188-193. 25. Brass P, Hellmich M, Kolodziej L, Schick G, Smith AF. Ultrasound guidance versus anatomical landmarks for subclavian or femoral vein catheterization. Cochrane Database of Systematic Reviews 2015;1:CD011447. |
| Other data access-related reason  (n=4) | 1. Donaldson JS, Norman JT, Morello FP, Saker MC. Pediatric vascular access. Seminars in Interventional Radiology 1998;15:315-323. 2. Kuo CC, Wu CY, Feng IJ, Lee WJ. Efficacy of Ultrasound-Guided Peripheral Intravenous Access: A Systematic Review and Meta-Analysis. Hu Li Za Zhi 2016; 63:89-101. 3. Gottlieb M, Long B. Ultrasound guidance for the placement of peripheral intravenous catheters. American family physician 2021;104:online 4. Caponnetto S, Martini U. The intravascular imaging technic with ultrasound. Cardiologia (Rome, Italy) 1991;36:165-168. |

**Table S4. Methodological quality assessment by AMSTAR-2 (a critical appraisal tool for systematic reviews of healthcare interventions)**

| **Reference** | **1** | **2** | **3** | **4** | **5** | **6** | **7** | **8** | **9** | **10** | **11** | **12** | **13** | **14** | **15** | **16** | **Overall confidence** |
| --- | --- | --- | --- | --- | --- | --- | --- | --- | --- | --- | --- | --- | --- | --- | --- | --- | --- |
| **Egan 2012** | **Yes** | **No** | **No** | **Yes** | **Yes** | **Yes** | **Partial Yes** | **Partial Yes** | **Yes** | **No** | **Yes** | **Yes** | **Yes** | **Yes** | **Yes** | **Yes** | Critically low |
| **Heinrichs 2012** | **Yes** | **No** | **Yes** | **Partial Yes** | **Yes** | **Yes** | **Yes** | **Partial Yes** | **Yes** | **No** | **Yes** | **Yes** | **Yes** | **Yes** | **No** | **Yes** | Critically low |
| **Kleidon 2021** | **Yes** | **Partial Yes** | **Yes** | **Partial Yes** | **Yes** | **Yes** | **Partial Yes** | **Partial Yes** | **Yes** | **Yes** | **Yes** | **Yes** | **Yes** | **No** | **No** | **Yes** | Critically low |
| **Liu 2014** | **Yes** | **No** | **Yes** | **Yes** | **Yes** | **Yes** | **Yes** | **Partial Yes** | **No** | **No** | **No MA** | **No MA** | **No** | **No** | **No MA** | **Yes** | Critically low |
| **Parker 2017** | **Yes** | **Partial Yes** | **No** | **Yes** | **Yes** | **No** | **Yes** | **Partial Yes** | **Yes** | **Yes** | **No MA** | **No MA** | **Yes** | **Yes** | **No MA** | **Yes** | Critically low |
| **Parker 2017** | **Yes** | **Partial Yes** | **No** | **Yes** | **Yes** | **No** | **Yes** | **Partial Yes** | **Yes** | **Yes** | **No MA** | **No MA** | **Yes** | **Yes** | **No MA** | **Yes** | Critically low |
| **Stolz 2015** | **Yes** | **No** | **No** | **Partial Yes** | **Yes** | **Yes** | **No** | **Partial Yes** | **No** | **No** | **Yes** | **No** | **No** | **Yes** | **No** | **Yes** | Critically low |
| **Tran 2021** | **Yes** | **No** | **No** | **Partial Yes** | **No** | **Yes** | **Yes** | **Partial Yes** | **Yes** | **No** | **Yes** | **Yes** | **Yes** | **Yes** | **Yes** | **Yes** | Critically low |
| **Tran 2022** | **Yes** | **No** | **No** | **Partial Yes** | **Yes** | **Yes** | **Yes** | **Partial Yes** | **Yes** | **No** | **Yes** | **Yes** | **No** | **Yes** | **Yes** | **Yes** | Critically low |
| **van Loon 2018** | **Yes** | **No** | **No** | **Partial Yes** | **Yes** | **Yes** | **No** | **Partial Yes** | **No** | **No** | **Yes** | **No** | **No** | **Yes** | **No** | **Yes** | Critically low |
| **Varndell 2018** | **Yes** | **No** | **No** | **Partial Yes** | **Yes** | **Yes** | **Yes** | **Partial Yes** | **Yes** | **No** | **No MA** | **No MA** | **Yes** | **No** | **No MA** | **Yes** | Critically low |
| **Ye 2022** | **Yes** | **No** | **Yes** | **Partial Yes** | **Yes** | **Yes** | **Yes** | **Partial Yes** | **Yes** | **No** | **Yes** | **Yes** | **No** | **Yes** | **No** | **Yes** | Critically low |

MA, Meta-analysis; RoB, Risk of bias.

Key:
ITEM DESCRIPTION

1. Did the research questions/inclusion criteria include the components of PICO?
2. Did the review contain an explicit statement that the review methods were established prior to the conduct of the review?
3. Did the review authors explain their selection of the study designs for inclusion in the review?
4. Did the review authors use a comprehensive literature search strategy?
5. Did the review authors perform study selection in duplicate?
6. Did the review authors perform data extraction in duplicate?
7. Did the review authors provide a list of excluded studies and justify the exclusions?
8. Did the review authors describe the included studies in adequate detail?
9. Did the review authors assess the RoB in studies that were included in the review?
10. Did the review authors report on the sources of funding for the studies included in the review?
11. If meta-analysis was performed, did the review authors use appropriate methods for statistical combination of results?
12. If meta-analysis was performed, did the review authors assess the potential impact of RoB in individual studies on the results of the meta-analysis?
13. Did the review authors account for RoB in individual studies when interpreting the results of the review?
14. Did the review authors provide a satisfactory explanation for, and discussion of, any heterogeneity observed in the results of the review?
15. If they performed quantitative synthesis, did the review authors investigate publication bias?
16. Did the review authors report any potential sources of conflict of interest, including any funding they received for conducting the review?

**Table S5. Certainty of evidence according to Grading of Recommendation Assessment, Development, and Evaluation (GRADE).**

| **Reference** | **Population** | **Number of studies** | **Number of participants (Imprecision)** | **Risk of bias (Trial quality)** | **Heterogeneity (Inconsistency)** | **Methodological quality*** | **Certainty** |
| --- | --- | --- | --- | --- | --- | --- | --- |
| ***Overall success*** | | | | | | | |
| Egan et al. 2012 | Adult and pediatric | 6 | 272 | Serious limitations | Serious limitations | Very serious limitations | ⨁⨁  Low |
| Kleidon et al. 2021 | Pediatric | 4 | 691 | Serious limitations | Serious limitations | Very serious limitations | ⨁⨁  Low |
| Stolz et al. 2015 | Adult and pediatric | 7 | 362 | Serious limitations | No serious limitations | Very serious limitations | ⨁⨁  Low |
| van Loon et al. 2018 | Adult | 8 | 1660 | Serious limitations | No serious limitations | Very serious limitations | ⨁⨁  Low |
| Ye et al.  2022 | Pediatric | 7 | 983 | Serious limitations | Serious limitations | Very serious limitations | ⨁⨁  Low |
| ***First-attempt success rate*** | | | | | | | |
| Kleidon et al. 2021 | Pediatric | 4 | 592 | Serious limitations | Serious limitations | Very serious limitations | ⨁⨁  Low |
| Tran et al.  2021 | Adult | 7 | 1603 | Serious limitations | No serious limitations | Serious limitations | ⨁⨁⨁  Moderate |
| Tran et al.  2022 | Adult | 7 | 527 | Serious limitations | No serious limitations | Very serious limitations | ⨁⨁  Low |
| van Loon et al. 2018 | Adult | 3 | 1284 | Serious limitations | No serious limitations | Very serious limitations | ⨁⨁  Low |
| Ye et al.  2022 | Pediatric | 7 | 878 | Serious limitations | Serious limitations | Very serious limitations | ⨁⨁  Low |
| ***Number of attempts*** | | | | | | | |
| Egan et al. 2012 | Adult and pediatric | 4 | 160 | Serious limitations | Serious limitations | Very serious limitations | ⨁  Very low |
| Heinrichs et al. 2012 | Adult and pediatric | 7 | 272 | Serious limitations | No serious limitations | Very serious limitations | ⨁⨁  Low |
| Stolz et al. 2015 | Adult and pediatric | 4 | 212 | Serious limitations | Serious limitations | Very serious limitations | ⨁⨁  Low |
| Tran et al.  2021 | Adult | 8 | 581 | Serious limitations | No serious limitations | Serious limitations | ⨁⨁⨁  Moderate |
| Tran et al.  2022 | Adult | 6 | 439 | Serious limitations | No serious limitations | Very serious limitations | ⨁⨁  Low |
| van Loon et al. 2018 | Adult | 4 | 230 | Serious limitations | Serious limitations | Very serious limitations | ⨁⨁  Low |
| Ye et al.  2022 | Pediatric | 5 | 682 | Serious limitations | Serious limitations | Very serious limitations | ⨁⨁  Low |
| ***Time*** | | | | | | | |
| Egan et al. 2012 | Adult and pediatric | 5 | 218 | Serious limitations | Serious limitations | Very serious limitations | ⨁⨁  Low |
| Heinrichs et al. 2012 | Adult and pediatric | 5 | 203 | Serious limitations | No serious limitations | Very serious limitations | ⨁⨁  Low |
| Stolz et al. 2015 | Adult and pediatric | 5 | 260 | Serious limitations | No serious limitations | Very serious limitations | ⨁⨁  Low |
| Tran et al.  2021 | Adult | 8 | 517 | Serious limitations | No serious limitations | Serious limitations | ⨁⨁⨁  Moderate |
| Tran et al.  2022 | Adult | 4 | 295 | Serious limitations | No serious limitations | Very serious limitations | ⨁⨁  Low |
| van Loon et al. 2018 | Adult | 4 | 230 | Serious limitations | Serious limitations | Very serious limitations | ⨁⨁  Low |
| Ye et al.  2022 | Pediatric | 6 | 719 | Serious limitations | Serious limitations | Very serious limitations | ⨁⨁  Low |
| ***Satisfaction*** | | | | | | | |
| Tran et al.  2021 | Adult | 3 | 187 | Serious limitations | No serious limitations | Serious limitations | ⨁⨁  Low |
| Tran et al.  2022 | Adult | 2 | 162 | Serious limitations | Serious limitations | Very serious limitations | ⨁  Very low |
| van Loon et al. 2018 | Adult | 2 | 135 | Serious limitations | No serious limitations | Very serious limitations | ⨁⨁  Low |

*NOTE: Pollock and colleagues proposed the application of the four AMSTAR critical domains for the 'Methodological quality’ domain. However, due to the recent publication of AMSTAR-2, the seven AMSTAR-2 critical domains were applied for the 'Methodological quality’ domain instead of the four previously published. The presence of 1 circled cross indicates ‘Very low’ certainty, and 4 indicate ‘High’ certainty.

**Table S6. Evidence map**

**Legend**

**Effect direction**

Improvement

No effect

No data

**GRADE certainty evidence**

High

Moderate

Low

Very low

| **Reference** | **Ultrasound-guided method** | | | | |
| --- | --- | --- | --- | --- | --- |
|  | **Overall success** | **First-attempt success rate** | **Number of attempts** | **Time** | **Satisfaction** |
| Egan et al.  2012 |  |  |  |  |  |
| Heinrichs et al.  2012 |  |  |  |  |  |
| Kleidon et al.  2021 |  |  |  |  |  |
| Liu et al.  2014 |  |  |  |  |  |
| Parker et al.  2017 |  |  |  |  |  |
| Parker et al.  2017 |  |  |  |  |  |
| Stolz et al.  2015 |  |  |  |  |  |
| Tran et al.  2021 |  |  |  |  |  |
| Tran et al.  2022 |  |  |  |  |  |
| van Loon et al.  2018 |  |  |  |  |  |
| Varnderll et al.  2018 |  |  |  |  |  |
| Ye et al.  2022 |  |  |  |  |  |

**Table S7. Characteristics of the randomized controlled trials which compare traditional versus US-guided method for SPC insertion and have been included in the previous meta-analyses.**

| **Study** | **Patients** | | | **Overall success rate,**  ***n(%)*** | **First-attempt success rate,**  ***n(%)*** | **Attempts, *mean (SD)*** | **Time (min),**  ***mean (SD)*** | **Satisfaction,**  ***mean (SD)*** | **Adverse effects,**  ***n(%)*** | **Hospital setting** | **Provider** | **DVA** |
| --- | --- | --- | --- | --- | --- | --- | --- | --- | --- | --- | --- | --- |
|  | **Age (y)** | **Total** | **Groups**  **C(n)**  **I(n)** |  |  |  |  |  |  |  |  |  |
| Aponte  2007 | >18 | 35 | Palpation (16)  US (19) | NA | 13 (81)  14 (74) | 1.3 (0.9)  1.4 (0.7) | 3.1 (3.8)  1.3 (0.5) | NA | NA | OR | Nurses | Yes |
| Avelar  2015 | 1-18 | 382 | Landmark (194)  US (188) | 178 (91.8)  161 (85.6) | NA | NA | NA | NA | Infiltration  C: 23 (12.9)  I: 34 (21.1)  Phlebitis  C: 7(3.9)  I: 2 (1.2) | Surgical unit | Nurses | No |
| Bair  2008 | <7 | 44 | Traditional (21)  US (23) | NA | 6 (29)  8 (35) | NA | NA | NA | NA | ED | Physicians and nurses | Yes |
| Bahl  2016 | >18 | 122 | Palpation (59)  US (163) | 33 (55.9)  48 (76.2) | NA | 1.71 (NA)  1.52 (NA) | 20.7 (NA)  15.8 (NA) | NA | NA | ED | Nurses | Yes |
| Benkhadra  2012 | <3 | 40 | Blind (20)  US (20) | 17 (85)  18 (90) | 7 (35)  17 (85) | 2.5 (1-6.5) ^†^  1 (1-1) ^†^ | 7 (3.2-12.8) ^†^  1.1(0.8-2.5) ^†^ | NA | NA | Anesthesia unit | NA | Yes |
| Bian  2021 | <3 | 144 | Conventional (72)  US (72) | 56 (77.8)  65 (90.3) | 27 (37.5)  45 (62.5) | 2 (1-3) ^†^  1 (1-2) ^†^ | 1.6 (0.9-2.9) ^†^  0.8 (0.6-1.6) ^†^ | NA | Infiltration  C: 2 (2.78)  I: 2 (2.78)  Pale skin  C: 0 (0)  I: 1 (1.4) | OR | Anesthetists | Yes |
| Bridey  2018 | >18 | 112 | Landmark (56)  US (56) | 39 (70)  37 (66) | 18 (33)  23 (41) | 2 (1-4) ^†^  2 (1-4) ^†^ | NA | 8 (7-9.5) ^†^  8 (7-9) ^†^ | Infiltration  C: 9(18)  I: 18 (34)  Accidental catheter removal  C: 6 (12)  I: 2 (4) | ICU | Nurses | Yes |
| Curtis  2015 | ≤3  3-16 | 92  191 | Standard (50)  US (42)  Standard (96)  US (95) | NA | 31 (62)  22 (52)  78 (81)  75 (79) | 1.76 (NA)  1.69 (NA)  1.26 (NA)  1.28 (NA) | 6.5 (10.3)  8.3(15.9) | NA | NA | ED | Nurses | No |
| Darvish  2011 | >18 | 25 | Standard (10)  ECO (15) | NA | NA | 2.3 (NA)  1.9(NA) | 28.1 (NA)  23.7 (NA) | 77.8 (NA)  93.3 (NA) | Pain  C: 6.6 (NA)*  I: 4.1 (NA)* | ED | Nurses | Yes |
| Doniger  2009 | <10 | 50 | Traditional (25)  ECO (25) | 20 (80)  16 (64) | NA | 3 (2-4) ^†^  1 (1-2.5) ^†^ | 14.4 (9.6)  6.3 (5.7) | NA | Arterial puncture  C: 1 (4)  I: 0 | ED | Physicians and nurses | Yes |
| Gopalasingam  2017 | <4 | 100 | Palpation (50)  US (50) | 42 (84)  50 (100) | 30 (60)  42 (84) | 1 (1-4) ^†^  1 (1-3) ^†^ | 1.7(0.2-13.4) ^†^  3.2 (0.6-14) ^†^ | NA | NA | OR | Anesthetists | No |
| Kerforne  2012 | >18 | 60 | Palpation (30)  US (30) | 11 (37)  21 (70) | NA | NA | 6.7 (3.3)  7.3 (5.1) | NA | NA | ICU | Nurses | Yes |
| Hanada  2017 | <4 | 102 | Landmark (51)  US (51) | 32 (63)  47 (92) | 26 (51)  46 (90) | 1.02 (NA)  1.31 (NA) | 2.5(1.9-4.1) ^†^  3.9 (3-5) ^†^ | NA | Arterial/nerve puncture, phlebitis, compartmental syndrome, tissue necrosis.  C: 0  I: 0 | OR | Anesthetists | Yes |
| McCarthy 2016 | >18 | 1189 | *Difficult access*  Landmark (94)  US (98)  *Moderate access*  Landmark (199)  US (202)  *Easy access*  Landmark (291)  US (305)  *Total*  Landmark (584)  US (605) | NA | 33 (35.1)  80 (81.6)  142 (71.4)  164 (81.2)  281 (96.6)  262 (85.9)  456 (78.1)  506 (83.6) | NA | 2.1 (NA) ^†^  3.5 (NA) ^†^  1.7 (NA) ^†^  3.3 (NA) ^†^  1.3 (NA) ^†^  2.7 (NA) ^†^ | NA | Infiltration  C*^1^*: 3 (3.2)  I*^1^*: 2 (2.1)  C^2^: 13 (6.5)  I^2^: 8 (4)  C^3^: 5 (1.7)  I^3^: 12 (3.9)  Arterial/nerve puncture  C*^1^*: 2 (2.1)  I*^1^*: 2 (2)  C^2^: 2 (1)  I^2^: 3 (1.5)  C^3^: 0 (0)  I^3^: 1 (0.3) | ED | ED technicians | Yes |
| Nishizawa  2020 | >18 | 60 | Landmark (30)  US (30) | NA | 12 (40)  21 (70) | 1.6 (0.5)  1.3 (0.45) | NA | NA | Infiltration  C:3 (28.6)  I: 3 (13.6) | ICU | Nurses | Yes |
| Pappas  2006 | >18 | 18 | Traditional (6)  US (12) | NA | NA | 3.2 (2.5)  1.7 (0.09) | 11.3 (8.5)  13.9 (13.2) | NA | Pain  C: 1.7 (0.5)*  I: 2.6 (2.4)* | OR, ICU, ED, post-anesthesia care unit, and mother-baby unit. | Anesthetists | Yes |
| River  2009 | NA | 47 | Traditional (21)  US (26) | 15 (72)  23 (87) | NA | 2 (NA)  1.5 (NA) | 22 (NA)  26(NA) | 3.4 (NA)  4.2 (NA) | NA | ED | Nurses | Yes |
| Stein 2009 | >18 | 59 | Traditional (31)  US (28) | 30 (97)  28 (100) | 10 (32.3)  11 (39.3) | 2.37 (0.77)  2.07(0.47) | 48.2 (28.7)  60.2 (26.8) | 7.04 (0.94)  7.96 (1.06) | NA | ED | Physicians | Yes |
| Vinograd  2019 | ≤3  3-18 | 100  67 | Traditional (50)  US (49)  Traditional (33)  US (33) | 44 (88)  47 (96)  30 (90.9)  33 (100) | 23 (46)  40 (81.6)  15 (45.5)  30 (90.9) | 2 (1-2) ^†^  1 (1-1) ^†^ | 28 (NA) ^†^  14 (NA) ^†^ | 8 (5-10) ^†^  10 (8-10) ^†^ | Phlebitis, infiltration, pain, leakage, bleeding, unintentional dislodgement and line occlusion  C: 19 (48)  I: 26 (40) | ED | Physicians, fellows and nurses | Yes |
| Vitto  2016 |  | 122 | Traditional (61)  US (61) | 34 (56)  61 (100) | NA | 2.16 (NA)  1.31 (NA) | NA | NA | NA | ED | Medical students | No |

C, Control; DVA, Difficult vascular access; ED, Emergency department; I, Intervention; ICU; Intensive care unit; IQR; Interquartile range; NA, Not available; OR, Operating room; SD, Standard deviation; SPC, Short peripheral catheter; US, Ultrasound; Y, Years

*Mean (SD) of pain measured on a scale of 0-10.

^†^Median (IQR)

^1^Difficult access group

^2^Moderate access group

^3^Easy access group

Note: The Costantino and colleagues (2010) study has been included in two previous systematic reviews (Stolz and colleagues -2015- and Tran and colleagues -2021-). However, it has been excluded of this table because their no exposed group is not defined as traditional peripheral intravenous access.

Note: Satisfaction was measured on a scale of 0-10 in all studies, although Darvish and colleagues represented the mean in percentage.

**Table S8. Study overlaps in included systematic reviews and meta-analyses.**

|  | Egan  2012 | Heinrichs  2012 | Liu  2014 | Parker  2017 | Parker  2017 | Stolz  2015 | Varndell  2018 | Van loon  2018 | Kleidon  2021 | Tran  2021 | Tran  2022 | Ye  2022 |
| --- | --- | --- | --- | --- | --- | --- | --- | --- | --- | --- | --- | --- |
| Aponte  2007 | x | x | x | x |  | x |  | x |  | x | x |  |
| Avelar  2015 |  |  |  |  |  |  |  |  | x |  |  | x |
| Bahl  2016 |  |  |  |  |  |  | x | x |  | x | x |  |
| Bair  2008 | x | x |  |  |  |  |  |  |  |  |  | x |
| Bauman  2009 |  |  |  |  |  |  |  | x |  | x |  |  |
| Benkhadra  2012 |  | x | x |  | x | x |  |  | x |  |  | x |
| Bian  2021 |  |  |  |  |  |  |  |  |  |  |  | x |
| Blaivas  2006 |  |  |  |  |  |  | x |  |  |  |  |  |
| Brannam  2004 |  |  |  |  |  |  | x |  |  |  |  |  |
| Bridey  2008 |  |  |  |  |  |  |  |  |  |  | x |  |
| Carter  2015 |  |  |  |  |  |  | x |  |  |  |  |  |
| Costantino  2005 |  |  |  |  |  | x |  | x |  | x |  |  |
| Costantino  2010 |  |  |  |  |  | x |  |  |  | x |  |  |
| Curtis  2015 |  |  |  |  | x |  |  |  | x |  |  | x |
| Doniger  2009 | x | x | x |  | x | x |  |  |  |  |  | x |
| Darvish  2011 | x | x |  |  |  |  |  |  |  |  |  |  |
| Feinsmith  2018 |  |  |  |  |  |  | x |  |  |  |  |  |
| Gopalasingam  2017 |  |  |  |  |  |  |  |  |  |  |  | x |
| Hanada  2017 |  |  |  |  |  |  |  |  | x |  |  | x |
| Ismailoglu  2015 |  |  |  |  |  |  | x | x |  |  | x |  |
| Kerforne  2012 | x | x | x |  |  | x |  | x |  | x | x |  |
| Mahler  2011 |  |  |  | x |  |  |  |  |  |  |  |  |
| McCarthy  2016 |  |  |  |  |  |  |  | x |  | x |  |  |
| Nishizawa  2020 |  |  |  |  |  |  |  |  |  |  | x |  |
| Oliveira  2016 |  |  |  |  |  |  | x |  |  |  |  |  |
| Pappas  2006 | x | x |  |  |  |  |  |  |  |  |  |  |
| River  2009 |  | x | x |  |  |  |  |  |  |  |  |  |
| Salleras-Duran  2016 |  |  |  |  |  |  | x |  |  |  |  |  |
| Stein  2009 | x | x | x |  |  | x |  | x |  | x |  |  |
| Vinograd  2019 |  |  |  |  |  |  |  |  | x |  |  | x |
| Vitto  2016 |  |  |  |  |  |  |  |  |  | x |  |  |
| Weiner  2013 |  |  |  |  |  |  | x |  |  | x | x |  |
| TOTAL | **7** | **9** | **6** | **2** | **3** | **7** | **9** | **8** | **5** | **10** | **7** | **9** |

**Supplemental Legends**

**Table S1.** PRISMA 2020 Checklist.

**Table S2.** Search strategy from database inception to 23rd February 2022.

**Table S3.** Excluded studies from the umbrella review with reasons.

**Table S4.** Methodological quality assessment by AMSTAR-2 (a critical appraisal tool for systematic reviews of healthcare interventions)

**Table S5.** Certainty of evidence according to Grading of Recommendation Assessment, Development, and Evaluation (GRADE).

**Table S6.** Evidence map.

**Table S7.** Characteristics of the randomized controlled trials which compare traditional versus US-guided method for SPC insertion and have been included in the previous meta-analyses.

**Table S8.** Study overlaps in included systematic reviews and meta-analyses.
